# Mental fatigue impairs physical activity, technical and decision-making performance during small-sided games

**DOI:** 10.1371/journal.pone.0238461

**Published:** 2020-09-09

**Authors:** Athos Trecroci, Gabriele Boccolini, Marco Duca, Damiano Formenti, Giampietro Alberti

**Affiliations:** 1 Department of Biomedical Sciences for Health, Università degli Studi di Milano, Milano, Italy; 2 Physical Performance & Sport Science Department, Atalanta Bergamasca Calcio, Bergamo, Italy; 3 Department of Biotechnology and Life Sciences (DBSV), University of Insubria, Varese, Italy; Universidade Federal de Juiz de Fora, BRAZIL

## Abstract

The aim of this study was to investigate the effects of mental fatigue on physical activity, technical and decision-making performance during small-sided games. Nine sub-elite soccer players were enrolled in the study. The players performed two small-sided games on two occasions within a crossover experimental design. Before each game, they underwent a mental fatiguing task (Stroop task) and a control task (documentary watching) in a randomized, counterbalanced order. Players’ physical activity, technical, and decision-making performance were obtained during small-sided games by GPS and video scouting. Results showed that distance in acceleration covered per min, negative passes, passing accuracy, and shot accuracy were *likely* impaired than control task after a mental fatiguing protocol. Decision-making performance of negative passes, passes accuracy, and dribbling accuracy resulted also *likely* decreased compared with control task. These findings demonstrated that mental fatigue impacted on technical, GPS-derived, and soccer-specific decision-making performance during SSG. In conclusion, avoiding cognitively demanding tasks before playing soccer-specific activities may be advisable to preserve players’ physical activity, technical, and decision-making skills.

## Introduction

Fatigue can be classified as mental or physical [1]. The former refers to a psychobiological state in which individuals manifest subjective, behavioural, and physiological alterations induced by prolonged or excessively demanding mental tasks [2]. According to Warm et al. [3] activities requiring a high vigilance are stressful as accompanied by hard mental work. From a behavioural perspective, mental fatigue has been shown to impact on muscle endurance, fatigue, and recovery during static shoulder abduction (to exhaustion) exercise [4], on motor performance (e.g. force) during submaximal hand-grip exercise [5], and on physical performance (e.g., time to exhaustion) during high-intensity cycling exercise [6]. Most of the literature investigated the effects of mental fatigue within a laboratory setting in which an individual is under limited changing ecological practice (e.g., physical, technical and tactical) constraints compared with team sports environment (i.e., soccer) [7]. Smith et al. [8] investigated the effects of mental fatigue on technical performance using Loughborough soccer passing and shooting tests. The authors observed that soccer players increased the number of errors in controlling and passing the ball as well as decreased the speed and accuracy of the shots after mentally fatiguing protocol (i.e., Stroop task). This result indicates that mental fatigue may qualitatively and quantitatively impair soccer-specific technical performance inducing slower and less precise shots [8]. Of note, the authors concluded that this negative effect of mental fatigue over the technical performance may be due to central cognitive process (altered perception or attention on unimportant stimuli) influencing the players’ ability to manage the ball [8]. However, this kind of conclusion appears limited as the information recorded from the Loughborough soccer passing and shooting tests do not reflect a game-based setting in which cognitive processes (decision-making actions) are highly demanding.

During a soccer match, players are constantly prompted to process a large array of continually changing information: the position of the ball, opponents, and teammates. From these, the resultant players’ decision-making performance is linked to the ability to adjust positioning based on knowledge of the surrounding environment. Thus, players are required players to be focused on more relevant information prior to put their response into practice [7]. This condition increases both mental and physical demands involving a negative consequence on individual’s technical and tactical performance [9–11]. In accordance, Smith et al. [9] instructed 12 young adult soccer players to select accurately and rapidly the appropriate action in response to specific sequences of play (e.g., 2 vs.1, 3 vs. 1, etc) during a film-based simulation. The players had to quickly and accurately respond within a spectrum of actions such as passing to a player, shooting or dribbling the ball. The result demonstrated that mental fatigue, induced by a 30-min paper version of a modified Stroop colour-word task, impaired players’ accuracy and response time. However, being film simulation a poor practical approach to detect the impact of mental fatigue, the obtained results should be corroborated in a real-world soccer specific setting (e.g., during small-sided game, SSG).

In soccer, SSG represents a typical training tool to impose physical activity, technical, tactical, and cognitive demands with high specificity. Indeed, SSGs are small versions of the formal game in which the format (number of players) and the size of the pitch may be adjusted to better engage players on desired physical, physiological, and cognitive stimuli [12]. SSG may represent a valid ecological alternative to assess the impact of mental fatigue on players’ performance, without compromising the outcome of a competitive match [11]. Badin et al. [11] showed that during a 5-a-side SSG (without goalkeepers), well-trained soccer players did not worsen their physical activity profile (e.g., high and very-high speed running, total distance) while decreased their technical performance (e.g., control errors, involvement, and tackle success) after a 30-min computerized Stroop task. The authors concluded that mental fatigue can negatively impact on technical performance during SSG rather than on physical activity profile. Conversely, Coutinho et al. [10] found that mental fatigue affected players’ physical activity profile during a 5-a-side SSG. Specifically, the authors found that mental fatigue group covered *likely* less total distance compared with the control group (not mentally fatigued). This might be due to a possible role of mental fatigue in limiting the exercise tolerance as previously suggested [6]. Moreover, the authors also found an altered tactical behaviour in the mental fatigue group compared with the control group. Specifically, it was observed a decrease in the longitudinal synchronization (i.e., vertical coordinated movement in the pitch by two teammates with same goal) during 5-a-side SSG (plus goalkeepers) and after completing a 30 min Stroop colour-word task. The authors also concluded that mental fatigue could affect the way players perceive and explore chances for a given action during a match [10]. As such, this may have implications on players’ soccer specific decision-making skills. Being such skills vital for soccer, their potential fluctuation would directly affect players’ performance. However, to the authors’ knowledge, only a study [13] investigated how and at which extent mental fatigue affected soccer-specific decision-making skills. Gantois et al. [13] demonstrated the negative effect of mental fatigue on the decision-making performance during a match-like setting (training match lasting 90-min) in professional players. The authors observed a performance decrease in the passing decision-making following a 30-min Stroop task condition. Practically speaking, an individual mentally fatigued was more prone to make bad decisions, such as passing the ball to a marked teammate. Of note, this information is of practical importance, warning coaches and practitioners to keep own players away from mentally fatiguing tasks (before a match) in the attempt to preserve passing decision-making performance. Although this result adds practical insights to the current knowledge, further information is warranted to assess if mental fatigue could also impair soccer-specific decision-making skills linked to a broader spectrum of technical performance (e.g., shots, dribbling, and tackles) and physical activity (GPS-derived metrics) under an ecological approach (during a match or SSG).

Therefore, the aim of this study was to test the effects of mental fatigue on physical, technical and decision-making performance in 5-vs-5 small-sided soccer games with goalkeepers. It was hypothesized that mental fatigue would impair physical activity (GPS-derived metrics), technical and decision-making performance.

## Material and methods

### Participants

Ten sub-elite soccer players (three defenders, four midfielders, and three attackers) from the same U19 team (from a semi-professional soccer academy) participated in the study. One player (a midfielder) dropped out at the beginning of the experimental period for external reasons. As a result, nine players (age, 17.6 ± 0.5 years; height, 1.81 ± 4.4 m; body mass, 68.5 ± 6.2 kg) completed the study. Exclusion criteria were lower limb injuries in the last year, inadequate training volume in the previous eight weeks (< four training sessions per week and at least a weekend match day), history of febrile illness, and consumption of medications for at least six months before the study. All participants were optimally accustomed to demanding training sessions and official matches before the study. The study was approved by the Ethical Committee of the University of Milan, in accordance with the Helsinki declaration. All participants and their parents were deeply informed about the purpose and potential experimental risks of the research. Parents or legal guardians provided the written informed consent before the investigation.

### Experimental approach

**Fig 1:**
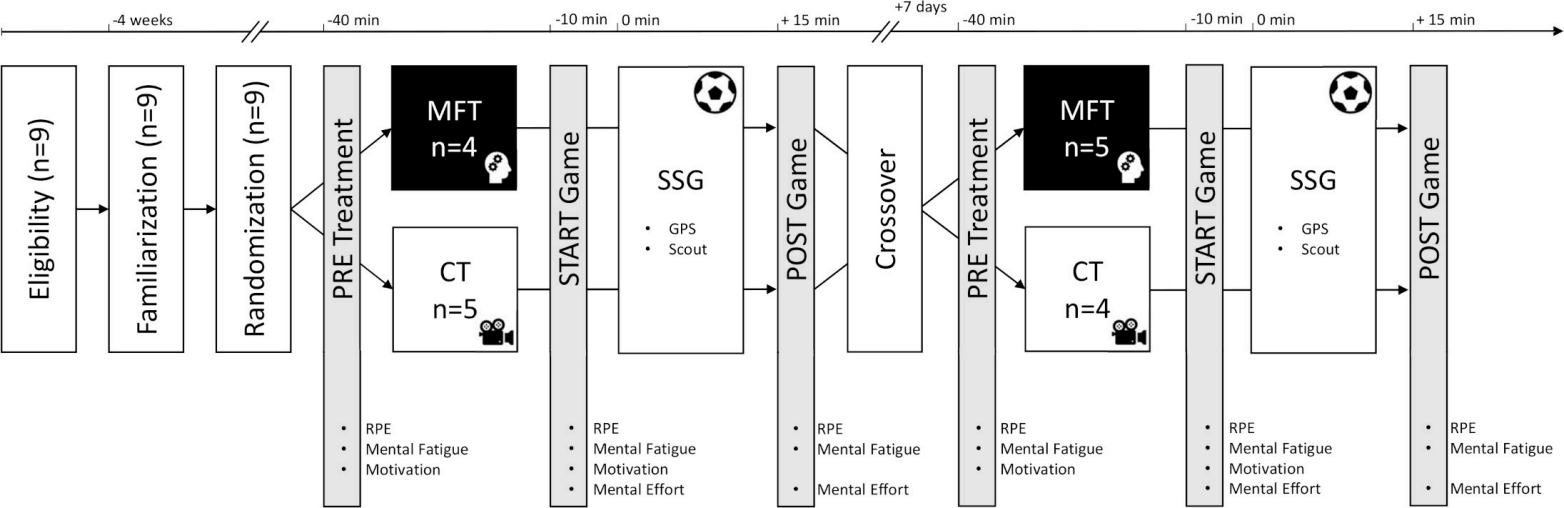
A schematic layout of the experimental design. MFT = mental fatigue group, CT = control group, RPE = rate of perceived exertion, SSG = small-sided games, GPS = global positioning system.

To investigate the effects of mental fatigue on soccer related performance during SSG this study employed a randomized counterbalanced cross-over design. Players were matched for their playing position and then randomly assigned to two groups. Group one performed a mental fatiguing task (MFT) on the first testing day and a control task (CT) on the second testing occasion, while group two underwent to CT on the first testing day and to MFT on the second testing day. A schematic representation of the design is showed in Fig 1. Additionally, two goalkeepers participated in this investigation only during SSGs, but they were not tested.

### Procedures

Two testing sessions, interspersed by one week, were carried out in the late afternoon during in season period before regular training. The players familiarized with all testing procedures before being involved in the experimental protocol. Specifically, within a four-weeks familiarization period (two sessions of ~1.5 h per week) [14], each player gained confidence with Borg’s CR10 Rating of Perceived Exertion (RPE) scale and 100-mm visual analogue scale (VAS) to assess perceptual responses (mental fatigue, mental effort, and motivation) [8, 11], and with Stroop task test [15]. Specifically, mental fatigue, mental effort and motivation represented subjective self-reported measures by VAS scale [16], which was labelled with “not at all fatigued” and “extremely fatigued”, “no effort at all” and “extreme effort”, “not at all motivated” and “extremely motivated”, respectively. The subjects were asked to consider their mental fatigue as a current state irrespective of what they have done before, whereas their mental effort as a perception of what they perceived during the previous task, and their motivation with respect to the following task. Participants were also instructed to maintain regular sleeping patterns (at least 8 h) and diet and to avoid caffeine, nicotine, alcohol, and mentally demanding tasks before subsequent testing sessions [11].

### Experimental protocol

Upon arrival at training ground, all players were assessed for their RPE, motivation (towards the upcoming treatment), mental fatigue, and physical fatigue (PRE treatment). Then, players were accompanied in quiet rooms where they were sat individually for 30 min. In this time-period, while players allocated to CT watched a documentary (as control condition), MFT were required to complete a Stroop Task [15] to induce mental fatigue. The Stroop Task was administered through “The Stroop Effect” App for Android systems using a smartphone with the same screen size for each player. As described in a recent review by Pageaux and Lepers [17] mental fatigue is induced by 30 min Stroop Task. Participants were instructed to complete the task as quickly and accurately as possible and were challenged to outperform their teammates. After completion of either Stroop task or documentary watching, players were again assessed for their RPE, motivation (towards the upcoming SSG with a 4-a-side format + 1 wildcard), mental effort (referred to the completed task), and mental fatigue (START game).

Of note, to maintain a balanced game situation, a certified soccer coach (with UEFA B license) played as a wildcard (an additional in-field player) in all SSGs. Before each SSG, all participants performed an 8-min-long technical warm up in a 10×10 m space. This standardized warm-up consisted of 3×2 min passing blocks and 2×1 min dynamic stretching periods [11]. The SSGs with goalkeepers were subdivided into 2 halves of 7 min each with 1 min halftime. The field size was double box (32×40 m) and the rules were the same as the regular soccer game, except for the offside rule, which was not implemented. When the ball came out the field, play started from goalkeeper. During all sessions, the matches were supervised by two operators instructed not to provide incitement or external support. The physical activity of the players during matches was assessed using GPS technology with an operational sampling frequency of 10 Hz (K-Sport GPS, Montelabate, Perugia, Italy) [18]. Each device was added to a tight vest with the GPS placed right between players’ scapulae. All GPS devices were previously turned on to favour an optimal acquisition of satellite signals. To ensure a reduced inter-unit variability, the players wore the same GPS device throughout the experimental protocol. For further details, please refer to the procedures adopted by Trecroci et al. [19]. Kinematic (total distance, distance per min, distance at speed > 15 km/h, and equivalent distance that is theoretically the distance covered at a steady on grass by the total energy expenditure over the session), mechanical (accelerations < 2 m/s^2^ and deceleration < -2 m/s^2^), metabolic loads (average metabolic power) were recorded and obtained to assess physical activity during SSGs. Matches were recorded using a video camera (Sony HD Video Recording HDRCX405, Tokyo, Japan) and analyzed with Kinovea software (http://www.kinovea.org/). The derived video footage was analysed by two football coaches with UEFA B license to scout each player’s technical and decision-making performance. Passing accuracy (successful/negative/total), tackling success (successful/negative/total) were calculated as percentages with respect to the total number performed by each player [11]. Successful intercepts were defined as gaining possession from an opponent’s pass, whereas unsuccessful intercepts were counted as control errors. Additionally, due to the presence of goalkeepers in the present study, it was possible to determine shots accuracy (successful/negative/total), calculated as percentages. Decision-making ability has been investigated using standardized coding criteria previously adopted [20, 21]. Passing, shooting, and dribbling decision-making skills were assessed. The quality of each decision was analyzed by the same experienced coaches and deemed as appropriate or inappropriate decisions. Inter-rater reliability from *kappa* (*k*) statistics revealed acceptable ranges of agreement for technical performance (0.870 < *k <* 1) and decision-making skills (0.866 < *k <* 1). After the SSG, players reported their RPE, mental effort (referred to the completed SSG), and mental fatigue (POST game).

### Statistical analyses

Data distribution was verified by the Shapiro-Wilk’s test. A paired *t*-test was performed to examine potential starting differences between MFT and CT at PRE. A two-way repeated measure analysis of variance (ANOVA RM) was used to detect possible interactions (Time x Intervention) and main effects of time and group between MFT and CT for perceptual responses (RPE and mental fatigue) between PRE, START, POST timepoints, and between START and POST timepoints (e.g. mental effort). In case of significance, Bonferroni post-hoc analysis was used. Within- and between-group comparisons were also performed using effect size (ES). The corresponding Cohen’s *d* ES was calculated and classified as trivial (ES < 0.2), small (0.2–0.5) moderate (0.5–0.8) and large (ES > 0.8). A significance level of 0.05 was chosen. The analysis was performed using the IBM SPSS® Statistics software (v. [21], New York, U.S.A.). To examine the practical relevance of the difference between MFT and CT in the scouting, decision-making, and GPS outcomes, the magnitude-based inference (MBI) was adopted [22]. The MBI allows to qualify and quantify the interpretation of the mean difference linked to a smallest worthwhile change (SWC [0.2 multiplied by the between-subject standard deviation]) [22]. For between-group comparisons, using a confidence interval of 90%, the chance for MFT of being lower, similar or higher to the CT for each variable was assessed qualitatively as follows: <1%, almost certainly not; 1–5% very unlikely; 5–25%, unlikely; 25–75%, possible; 75–95%, likely; 95–99%, very likely; and >99%, almost certain. If the chance of having greater and poorer differences was both >5%, the true difference was assessed as unclear [22]. MBI calculations and interpretations were used on a customized spreadsheet available at www.sportsci.org/index.html.

## Results

**Fig 2:**
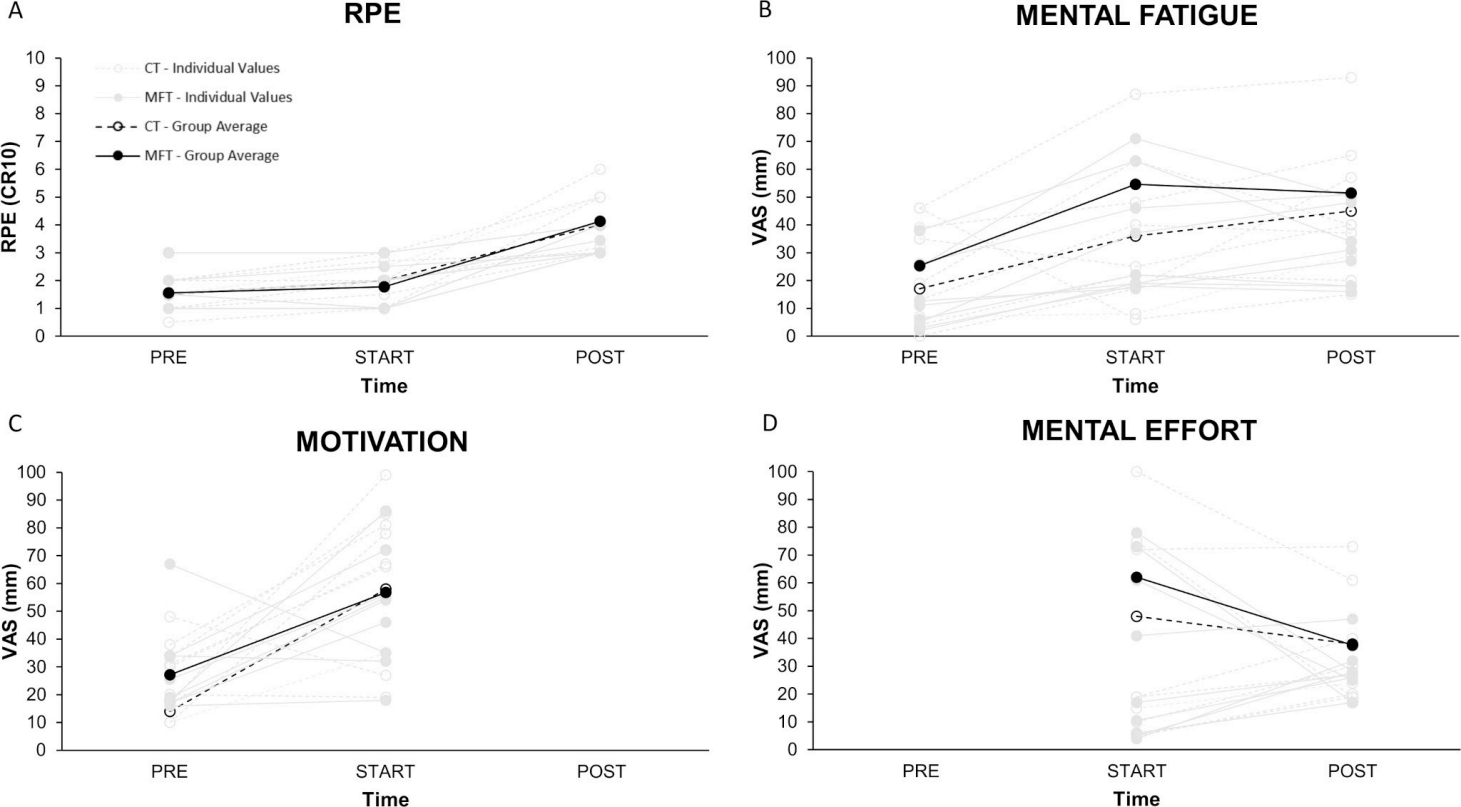
Perceptual responses of RPE (panel A), mental fatigue (panel B), motivation (panel C) and mental effort (panel D) throughout the experimental period (PRE, START, and POST). RPE = rate of perceived exertion.

No significant differences were observed in RPE (p = 0.347), mental fatigue (p = 0.062) at PRE. The Fig 2 shows the perceptual responses for each of the interventions throughout the experimental period. For RPE, two-way ANOVA RM revealed a significant interaction for RPE (F_2,16_ = 5.47; p = 0.015), a significant main effect of time (F_2,16_ = 49.43; p < 0.0001), and a non-significant main effect of group (p = 0.424). Multiple comparison analysis reported that both MFT (ES = 2.72, p < 0.0001) and CT (ES = 1.76, p < 0.0001) increased their RPE values from START to POST with a *large* effect, respectively. As regards between-group comparison (MFT versus CT), RPE values presented differences with *small* (ES = 0.32) and *moderate* (ES = 0.77) effects at START and POST, receptively. For mental fatigue, it was found a significant interaction (F_2,16_ = 3.671; p = 0.049), a significant main of effect of time (F_2,16_ = 19.46; p < 0.0001), and a significant main effect of group (F_1,8_ = 30.12; p < 0.001). Multiple comparison analysis reported that MFT increased their mental fatigue with a *large* effect (ES = 1.88, p = 0.001) from PRE to START, while decreased with a *trivial* effect (ES = 0.17, p = 0.99) from START to POST. While, CT increased their mental fatigue with a *small* effect (ES = 0.38, p = 0.99) from PRE to START, and with a *large* effect (ES = 0.96, p = 0.44) from START to POST. As regards between-group comparison (MFT versus CT), mental fatigue values presented differences with a *large* effect (ES = 2.84 and ES = 1.51) at START and POST, respectively. For motivation, it was found a non-significant interaction (p = 0.930), a significant main effect of time (F_1,8_ = 9.54, p = 0.01), and a non-significant main effect of group (p = 0.82). Multiple comparison analysis reported that both MFT (ES = 1.42, p = 0.001) and CT (ES = 1.44, p = 0.001) increased their motivation values from PRE to START with a *large* effect, respectively.

As regards between-group comparison (MFT versus CT), motivation presented differences with a *trivial* (ES = 0.07) effect. For mental effort, it was found a significant interaction (F_1,8_ = 12.39; p = 0.008), a non-significant main effect of time (p = 0.31), and a significant main effect of group (F_1,8_ = 26.42, p < 0.001). Multiple comparison analysis reported that MFT decreased mental effort with a *large* effect (ES = 1.07, p = 0.03), while CT increased mental effort with a *large* effect (ES = 2.24, p = 0.17). As regards between-group comparison (MFT versus CT), mental effort presented differences with a *large* effect (ES = 2.71) at START and a *moderate* effect (ES 0.79) at POST.

### Physical activity profile

Detailed information on the profile of GPS-derived metrics are shown in Table 1. In MFT, acceleration (m/min) and the sum of acceleration and deceleration (m/min) were *likely* lower than CT, while equivalent distance (%) presented *possibly* lower values than CT. Regarding the remaining of the GPS-related variables, *unclear* changes were observed between MFT and CT (Table 1).

**Table 1:** The overall physical activity variables (derived from GPS) expressed as mean ± SD.

Physical activity	MFT	CT	Mean difference as percentage	ES (90% CI)	Qualitative inference
Total distance (m)	949.3 ± 372.5	1050.6 ± 391.8	9.6%	-0.21 (-1.24, 0.83)	24/25/50 *Unclear*
Distance High Intensity Acceleration (m)	67.5 ± 28.5	78.7 ± 28.4	14.2%	-0.41 (-1.53, 0.71)	17/20/63 *Unclear*
Distance High Intensity Deceleration (m)	73.6 ± 33.9	83.2 ± 30.5	11.5%	-0.36 (-1.52, 0.81)	20/21/60 *Unclear*
Acceleration + Deceleration (m)	141.2 ± 62.4	162.0 ± 58.7	12.8%	-0.38 (-1.53, 0.76)	19/20/61 *Unclear*
Distance Velocity > 15km/h (m/min)	4.7 ± 2.2	5.3 ± 2.1	10.4%	-0.15 (-0.73, 0.43)	15/42/44 *Unclear*
Distance Metabolic Power > 20 (m)	211.5 ± 101.1	227.3 ± 95.9	6.9%	-0.16 (-1.18, 0.87)	27/26/47 *Unclear*
Relative Distance (m/min)	93.6 ± 13.1	94.6 ± 9.0	0.9%	-0.14 (-1.02, 0.74)	25/30/45 *Unclear*
Acceleration (m/min)	6.6 ± 1.3	7.1 ± 0.7	7.6%	-0.71 (-1.51, 0.09)	3/10/86 *Likely*
Deceleration (m/min)	7.1 ± 1.3	7.5 ± 0.8	5.8%	-0.54 (-1.36, 0.29)	7/17/77 *Unclear*
Acceleration + Deceleration (m/min)	13.7 ± 2.6	14.7 ± 1.6	6.7%	-0.64 (-1.45, 0.17)	4/13/83 *Likely*
Average Metabolic Power (W)	9.2 ± 1.2	9.4 ± 0.8	1.5%	-0.20 (-1.13, 0.73)	22/28/50 *Unclear*
Equivalent Distance (m)	1213.7 ± 474.3	1348.3 ± 495.2	9.9%	-0.22 (-1.27, 0.82)	24/25/51 *Unclear*
Equivalent Distance (%)	28.0 ± 4.4	28.7 ± 3.5	2.2%	-0.19 (-0.56, 0.18)	4/48/48 *Possibly*

SD = standard deviation, MFT = mental fatiguing task, CT = control task, ES = effect size, CI = confidence intervals.

### Technical performance

The overall technical performance is shown in Table 2. In MFT, negative passes resulted *likely* higher than CT, while passing accuracy and shot accuracy presented *likely* lower values than CT. Regarding the remaining of the technical variables, *unclear* changes were observed between MFT and CT (Table 2).

**Table 2:** The overall technical performance variables expressed as mean ± SD.

Technical performance	MFT	CT	Mean difference as percentage	ES (90% CI)	Qualitative inference
Possessions (n)	8.4 ± 3.9	8.0 ± 4.0	-5.5%	0.08 (-0.54, 0.69)	36/43/21 *Unclear*
Negative passes (n)	2.3 ± 1.1	1.5 ± 1.2	-50.0%	0.67 (0.01, 1.32)	89/9/2 *Likely*
Positive passes (n)	11.2 ± 3.3	12.8 ± 3.4	12.9%	-0.44 (-1.25, 0.37)	9/21/70 *Unclear*
Total passes (n)	13.5 ± 3.2	14.3 ± 3.0	5.4%	-0.25 (-1.11, -0.61)	18/28/54 *Unclear*
Passes Accuracy (%)	82.0 ± 9.2	88.2 ± 10.4	7.0%	-0.49 (-1.16, 0.17)	4/17/78 *Likely*
Negative shots (n)	1.1 ± 1.0	1.1 ± 1.2	0%	0.25 (-1.21, 1.71)	53/21/26 *Unclear*
Positive shots (n)	2.1 ± 2.0	3.7 ± 3.2	44.1%	-0.26 (-1.14, 0.63)	17/28/55 *Unclea*r
Total shots (n)	3.2 ± 2.7	4.8 ± 4.3	34.0%	-0.31 (-1.09, 0.48)	13/27/60 *Unclear*
Shot accuracy (%)	51.5 ± 41.9	81.7 ± 15.9	36.9%	0.01 (-1.01, 1.03)	36/29/35 *Likely*
Control Error (n)	1.8 ± 1.1	1.6 ± 1.4	-13.3%	0.27 (-0.35, 0.89)	58/32/10 *Unclear*
Negative Tackles (n)	1.8 ± 1.45	1.5 ± 1.4	21.4%	0.25 (-0.50, 1.00)	55/31/14 *Unclear*
Positive tackles (n)	2.1 ± 1.9	2.2 ± 1.7	5.0%	-0.48 (-1.35, 0.39)	9/19/72 *Unclear*
Total tackles (n)	3.8 ± 2.52	3.77 ± 2.1	-2.9%	0.10 (-0.50, 0.69)	38/43/19 *Unclear*
Tackle Success (%)	46.6 ± 27.1	49.1 ± 34.4	4.9%	-0.43 (-1.12, 0.26)	6/21/73 *Unclear*

SD = standard deviation, MFT = mental fatiguing task, CT = control task, ES = effect size, CI = confidence intervals.

### Soccer-specific decision-making performance

Table 3 shows the overall decision-making data for both interventions. In MFT, decision-making negative passes resulted *likely* higher than CT, while decision-making passes accuracy and decision-making dribbling accuracy presented *likely* lower values than CT. Regarding the rest of the decision-making variables, *unclear* changes were observed between MFT and CT (Table 3).

**Table 3:** The overall decision-making performance variables expressed as mean ± SD.

Decision-Making performance	MFT	CT	Mean difference as percentage	ES (90% CI)	Qualitative inference
Decision-making negative passes (n)	2.4 ± 1.1	1.5 ± 1.2	-57.1%	0.74 (0.05, 1.44)	91/7/2 *Likely*
Decision-making positive passes (n)	11.1 ± 3.4	12.8 ± 3.4	13.7%	-0.48 (-1.31, 0.35)	8/19/73 *Unclear*
Decision-making total passes (n)	13.5 ± 3.2	14.3 ± 3.0	5.4%	-0.25 (-1.11, 0.61)	18/28/54 *Unclear*
Decision-making passes accuracy (%)	81.5 ± 9.5	88.2 ± 10.4	7.5%	-0.53 (-1.23, 0.16)	4/16/80 *Likely*
Decision-making negative shots (n)	3.1 ± 2.6	4.5 ± 4.0	31.7%	-0.32 (-1.11, 0.48)	13/27/61 *Unclear*
Decision-making positive shots (n)	0.1 ± 0.3	0.2 ± 0.4	50.0%	-0.86 (-1.38, -0.30)	53/21/26 *Unclear*
Decision-making total shots (n)	3.2 ± 2.7	4.7 ± 4.3	32.5%	-0.31 (-1.09, 0.48)	13/27/60 *Unclear*
Decision-making shots accuracy (%)	86.6 ± 33.1	97.3 ± 5.2	10.9%	0.00 (-1.33, 1.33)	39/22/39 *Unclear*
Decision-making negative dribbling (n)	1.6 ± 1.2	0.8 ± 1.2	-87.5%	-0.23 (-0.24, 0.78)	19/28/53 *Unclear*
Decision-making positive dribbling (n)	2.2 ± 2.2	3.6 ± 3.7	39.3%	-0.34 (-1.16, 0.48)	12/25/63 *Unclear*
Decision-making total dribbling (n)	3.8 ± 3.2	4.5 ± 3.7	14.6%	-0.09 (-0.76, 0.58)	22/40/38 *Unclear*
Decision-making dribbling accuracy (%)	41.3 ± 32.2	75.3 ± 35.5	45.1%	-0.69 (-1.52, 0.13)	4/10/86 *Likely*

SD = standard deviation, MFT = mental fatiguing task, CT = control task, ES = effect size, CI = confidence intervals.

## Discussion

The main findings of this study were that mental fatigue affected the number of negative passes, passing accuracy, shot accuracy, distance in acceleration covered per min, decision-making skills linked to the number of negative passes, passing accuracy and dribbling accuracy. According to our previous hypothesis, mental fatigue impacted on physical activity, technical, and soccer-specific decision-making performance during SSG. The present perceptual responses also demonstrated that players in MFT increased their subjective rating (VAS score) of mental fatigue compared with CT, supporting the notion that individuals can be mentally fatigued after a 30-min cognitively demanding task [6, 9, 23].

The present study is the first to document the detrimental effect of mental fatigue on soccer-specific decision-making skills during SSG. To the extent of the authors’ knowledge, to date, only the study of Gantois et al. [13] examined the effects of mental fatigue on decision-making skills (i.e., on passing decision-making skill) under an ecological environment. The authors found that passing decision-making performance in male professional soccer players declined during a 90-min training match and after a prolonged (15–30 min) cognitive task. According to our results, it appears that mental fatigue can negatively influence not only passing decision-making skill, but also dribbling. Potential alterations in players’ perception (e.g., due to teammates and opponents’ position, and anticipatory cues to react to) may explain such skill impairment [13]. For instance, if a mentally fatigued player decides to dribble his or her opponent, he or she might be more prone to make a bad decision, as his or her dribbling will likely not create space for teammates or scoring opportunities. Specifically, the player in possession of the ball is likely to explore information related with the body position of the direct opponent. However, the occurrence of mental fatigue can lead the player to use less relevant information affecting the decision-making process. This provides additional and extended knowledge on the role of demanding-cognitive tasks on players’ ability to make accurate decision when performing on the pitch.

As regards technical performance, the present findings are partially supported by Badin et al. [11]. Specifically, the authors found that, in well-trained soccer players, MFT, compared to a control condition, led to *very likely* lower percentage of possession, *very likely* higher number of control errors, *likely* lower percentage of tackle success, and *possibly* lower percentage of passing accuracy. Furthermore, our results demonstrated that shot accuracy together with the number of negative passes were also affected by mental fatigue. Hereby, during SSG, an increased perception of effort could have affected technical performance in already mentally fatigued players to a greater extent than their peers in the control condition [8]. Accordingly, MFT exhibited higher values of mental effort than CT both at START and POST time points. This result indicates that mental fatigue, which contributed to increase the initial perception of effort, impaired players’ technical ability favouring the persistence of an elevated mental effort throughout the SSG. This consideration is reinforced by the fact that no differences were observed in motivation immediately prior to SSG between MFT and CT.

In the present study, we observed a detrimental effect of mental fatigue on the players’ physical activity, derived by GPS. After Stroop task, players covered *likely* lower distance at accelerating, and *possibly* lower equivalent distance than CT; however, this appears to be in contrast with Badin et al. [11]. Although the authors did not control for specific variable such as equivalent distance, they did not find any change in acceleration after a cognitively demanding task, induced by Stroop task, during SSG. A likely explanation of such contradictory finding may be attributed to the different assessment of acceleration. Indeed, while the authors assessed the number of accelerations, we used the distance covered at accelerating. Moreover, they considered accelerations >2.78 m/s^2^, while we considered those >2 m/s^2^. The fact that MF could negatively impact players’ ability to accelerate for a certain distance may be of practical importance and reinforce the advice that highly demanding-cognitive task should be avoided before a soccer-specific activity (e.g., SSG or match-play) [13]. On one hand, it might be argued that if mentally fatigued players lose more balls and fail more dribbles, they would be expected to perform more accelerations to compensate the decrease of their technical performance. On the other hand, it may be speculated that mental fatigue may also induce players’ tiredness or an aversion (perhaps linked to an increased perceived effort) likely making the players less prone to increase their physical activities [24] (e.g., number of accelerations) on the pitch. In support to this, the present perceptual responses (RPE) were higher in MFT than CT, suggesting that mental fatigue, linked to increased perceived effort, may have played a role in affecting physical activity during SSG. However, caution should be applied when interpreting such results, because of the dearth of comparative data and evidences in literature. Additional studies are warranted to get additional and extended knowledge on how mental fatigue affects players’ physical activity on the field. Summing up, the present results provide coaches and practitioners with additional information on the impact of mental fatigue on their players’ physical activity as well as their technical and decision-making performance during SSG. This may have relevant practical implications in preparing for the competition, where coaches should be concerned with any cognitively fatiguing task their players may be engaged in. It is particularly true in the moments preceding the competition itself, during which players may carry out different routines (e.g., smartphone usage and listening music). Indeed, the use of certain specific mobile games may represent a cognitively fatiguing task with a potential negative effect on brain response [25].

This study presents three main limitations that should be clearly acknowledged. First, given the players’ competitive level (sub-elite), generalizations on players with diverse level of play (elite) should be made with caution. Indeed, as players of higher level perform better in cognitive tasks [21], elite players might be less susceptible to mental fatigue [23], even though it remains unclear by the existing literature. Second, mental fatigue was induced by a prolonged computer-based cognitive task, which does not reflect the real-world stimuli anticipating a soccer activity (e.g., players interviews, player-coach interactions). Moreover, considering the individual responses, it might be possible that the present protocol did not induce mental fatigue in all players. Future studies should embed mental fatiguing protocol based on more specific pre-match activities [23] also capable to induce mental fatigue in sub-elite professional soccer players. Third, considering the current sample size, caution should be applied when interpreting our results. Nevertheless, the adopted design (crossover) would partially obviate the small size of the sample [19].

## Conclusions

Results from the present study demonstrated that mental fatigue impacted on physical activity (GPS-derived metrics), technical, and decision-making performance during SSG. Players are encouraged to avoid cognitively demanding tasks before engaging with soccer-specific activities to improve their readiness in terms of technical, physical and decision-making skills.

## Supporting information

S1 Dataset (SAV)
